# Interest of Follow-Up Radiological Imaging in Patients with Pyogenic Vertebral Osteomyelitis

**DOI:** 10.3390/jcm10122690

**Published:** 2021-06-18

**Authors:** Sophie Hecquet, Frank Verhoeven, Sébastien Aubry, Clément Prati, Daniel Wendling, Catherine Chirouze, Kévin Bouiller

**Affiliations:** 1 Department of Rheumatology, Besançon University Hospital, 25020 Besançon, France; shecquet@chu-besancon.fr (S.H.); fverhoeven@chu-besancon.fr (F.V.); cprati@chu-besancon.fr (C.P.); dwendling@chu-besancon.fr (D.W.); 2 PEPITE EA4267, FHU INCREASE, Bourgogne Franche-Comté University, UFR Santé, 25020 Besançon, France; 3 Department of Osteoarticular Radiology, Besançon University Hospital, 25020 Besançon, France; saubry@chu-besancon.fr; 4 EA 4266, EPILAB, Pathogens and Inflammation, Bourgogne Franche-Comté University, UFR Santé, 25020 Besançon, France; 5 Department of Infectious Disease, Besançon University Hospital, 25020 Besançon, France; cchirouze@chu-besancon.fr; 6 UMR CNRS, Bourgogne Franche-Comté University, 25020 Besançon, France

**Keywords:** spondylodiscitis, pyogenic vertebral osteomyelitis, septic discitis, MRI, CT-scan

## Abstract

No recommendations are established for monitoring pyogenic vertebral osteomyelitis (PVO). Thus, the realization of systematic follow-up radiological imaging is controversial. The objective of this study was to evaluate the interest in follow-up radiological imaging in patients with PVO. We conducted a retrospective cohort analysis of patients with PVO who had both baseline and follow-up radiological imaging. We classified the follow-up images into two groups, improvement/stability, and deterioration, compared with the baseline data. For each patient, we compared their radiological imaging follow-up to their clinical-biological condition assessed at the same time. Eighty-six patients were included. The mean age was 68 years (±13). A total of 99 radiological imaging examinations at diagnosis and at follow-up were analyzed, 69 Magnetic Resonance Imaging (MRI), and 30 Computerized Tomography (CT scans). The mean delay between the follow-up radiological imaging and clinical evaluation was 2.8 +/− 2.1 months. Of the 36 patients with clinical and biological recovery, 24 patients (67%) had improved radiological imaging and 12 patients (34%) had radiological worsening (new abscesses (*n* = 4), extension of soft tissue infiltration (*n* = 2) and/or epiduritis (*n* = 2) or appearance of new locations (*n* = 1)). Among the 50 patients considered as unhealed, on the contrary, radiological imaging showed an improvement in imaging in 39 patients (78%) and a worsening in 11 patients (22%). Our study showed that there was no correlation between the clinical condition of patients and their follow-up radiological imaging in the context of PVO.

## 1. Introduction

Pyogenic vertebral osteomyelitis (PVO), also called spondylodiscitis, septic discitis, and spinal osteomyelitis [1], represents 2% to 4% of osteoarticular infections [2]. Their incidence is steadily increasing due to the aging of the population and the rising number of people with immunocompromised conditions [3]. The diagnosis of PVO is often delayed despite the advances in imaging in recent years. Currently, magnetic resonance imaging (MRI) is the most efficient imaging technique for diagnosis in primary (by hematogenic origin) PVO, while [18F]FDG positron emission tomography (PET)/computed tomography (CT) is more accurate in secondary (post-surgical) PVO [4,5]. MRI is also the most reliable examination for the detection of complications, particularly medullar compression, and the detection of possible differential diagnoses, such as cancer [6].

The follow-up radiological imaging of patients with PVO is not standardized. It is, therefore, common that some physicians prescribe systematic follow-up MRI. Although some authors have suggested specific monitoring criteria in MRI [7], the correlation with the clinical course is discussed. Indeed, previous studies have shown that the abnormalities on MRI can persist for up to several months after clinical recovery or even worsen [6,8,9]. Thus, a study has highlighted the persistence of MRI anomalies up to 12 months after the introduction of an antibiotic therapy that has led to the clinical and biological recovery of a patient [10]. However, most data come from studies with relatively small patient populations [11]. In addition, little data are available on different radiological imaging follow-up modalities [12].

The objective of this study was to evaluate the interest in a follow-up of radiological imaging in patients with PVO.

## 2. Materials and Methods

We conducted a retrospective analysis of patients with PVO between January 2010 and August 2019 from the University Hospital of Besançon (France). The diagnosis of PVO was confirmed by a positive microbiologically sample (identification of pathogens in the tissue acquired by biopsy or surgery, or blood culture) and typical radiological features (with 1.5 Tesla MRI (AREA, Siemens Medical systems, Erlangen, Germany) or Computerized Tomography (Somatom Definition AS+, Siemens Medical systems, Erlangen, Germany)). The duration of follow-up was defined by the date of the patient’s post-antibiotic consultation, for which a clinical examination, a biological assessment, and radiological imaging were performed.

Only patients with the same radiological imaging (MRI or CT scan) at diagnosis and follow-up were analyzed. The images were analyzed by two different physicians blind to clinical and biological data. Follow-up images were compared to those taken at diagnosis and were classified into two groups: improvement/stability group or deterioration group. The following radiological imaging parameters were assessed: soft tissue infiltration, epidural, para-vertebral and intra-muscular abscesses, and epiduritis. Patients were classified in the deterioration group when worsening of at least one of the radiological imaging parameters was observed; otherwise, they were classified in the improvement/stability group. Then each follow-up radiological imaging was compared to the clinical-biological status of the patient assessed at the time of the examination. To assess the clinical status of patients, we retrospectively extracted clinical information from patients’ medical records. Patients were considered cured if they had recovered from their clinical condition prior to infection, and the CRP had to be less than 10 mg/L.

Statistical analysis. All variables were examined by univariate analysis using the chi-square or Fisher’s exact test, as appropriate. Continuous variables were analyzed by Student’s *t*-test. All statistical tests were two-tailed, and *p* < 0.05 was considered statistically significant. Statistical analyses were computed by SPSS 22.0 (IBM, Armonk, NY, USA).

## 3. Results

We included 86 patients. The mean age was 68 years (±13 years), 58 were men. Among these patients, 21 had a history of spinal surgery, 9 of them with osteosynthesis. The most frequently reported germs were methicillin-sensitive *Staphylococcus aureus* (34%), gram-negative bacilli (20%), and *Streptococcus* (19%). The level of spinal involvement was predominantly lumbar (Table 1).

The mean delay between diagnosis and follow-up radiological imaging was 2.77 ± 2.1 months. Initially, 84 (98%) patients presented with spinal pain, and 29 (34%) had a neurological deficiency, whereas at the follow-up assessment, there were 21 (24%) patients with spinal pain (*p* < 0.001) and 10 (12%) with neurological impairment (*p* < 0.001). The average CRP at diagnosis was 65 mg/L versus 26 mg/L at follow-up (*p* = 0.01) (Table 2).

A total of 99 diagnosis images and 99 follow-up images from 86 patients were analyzed, 69 MRIs, and 30 CT scans.

Only soft tissue infiltration was significantly less frequent on follow-up radiological imaging than on diagnostic imaging, either on CT scans (17 vs. 9; *p* = 0.037) or MRI (56 vs. 38; *p* < 0.01). Epiduritis was significantly less frequent at follow-up, only with MRI (38 vs. 23; *p* = 0.01) (Table 2).

Of the 36 patients with clinical and biological recovery, 24 patients (67%) showed an improvement of radiological images, and 12 patients (34%) showed radiological deterioration. Among the 50 patients considered as unhealed, on the contrary, radiological imaging showed an improvement in radiological lesions in 39 patients (78%) and a worsening in 11 patients (22%) (Table 3).

There was no significant difference between the healed and unhealed groups in the reduction/improvement of abscesses, soft tissue infiltration, and epiduritis. Soft tissue infiltration was the parameter with the greatest improvement between initial and follow-up radiological imaging in healed patients (*n* = 11, 45%). In unhealed patients, all parameters, abscess, epiduritis, and soft tissue infiltration had a similar reduction of approximately 30% (Table 3).

Although the results were not significant, we observed that patients considered cured had a slightly shorter diagnosis time than patients not cured (Table 3). However, we did not observe a correlation between radiological imaging deterioration and diagnosis time in patients considered to be uncured. Thus, in the unhealed patients with deteriorating imaging, the diagnosis time was 18 ± 11 days, whereas the diagnosis time was 52 ± 30 days when imaging was classified as improving/stable.

Among the 13 patients who had spinal surgery, five were operated on immediately following diagnostic radiological imaging due to neurological damage (spinal cord compression, cauda equina syndrome), four required abscess drains because of the increase or persistence of abscess without any associated neurological deficit. Of these four patients, only one was classified as completely healed. Two patients underwent a laminectomy, and two patients a corporectomy.

Of the 21 patients with a history of spinal surgery, six were considered cured, with only one image classified in the deterioration group. Among the 15 patients considered unhealed, 12 follow-up images showed the condition to be improving/stable, and three were worsening.

## 4. Discussion

Our results are consistent with those previously reported in the literature regarding PVO. Thus, our patients were mainly men; there was a predominance of staphylococci responsible for the infection and a higher frequency of lumbar involvement [13].

To our knowledge, this is one of the first studies comparing several follow-up radiological imaging modalities of patients with PVO with the clinical and biological status of the patients at the same time. Our study shows that there was no correlation between the patient’s clinical and biological status and his follow-up radiological imaging on average 3 months after first imaging, regardless if an MRI or a CT scan was performed.

Although the majority of previous studies suffer from their small sample size [8,9,10], our data support their results [8,9,14]. Thus, Kowalski et al. found no correlation between follow-up MRI and clinical status in two studies involving 79 and 33 patients [8,9]. Similarly, Carraghee et al. did not report any specific association between follow-up MRI results and a patient’s clinical status. In addition, this study showed that unnecessary surgeries could have been performed following the results of some follow-up MRIs [8,14]. This confirms that systematic follow-up radiological imaging should not be performed in patients with PVO [14,15].

Previous studies have reported that patients with PVO may have worsened bone and disc damage on MRI despite clinical improvement. In contrast, a soft tissue lesion tended to improve and was one of the first signs of improvement in MRI. It has also been shown that gadolinium contrast enhancement could persist for more than four months, although its intensity may be reduced. Contrast enhancement was undetectable after 2 years of follow-up [15,16].

The persistence of contrast enhancement on follow-up MRI despite clinical improvement has also been described [17,18]. The persistence of MRI abnormalities has been described up to 12 months after proper antibiotic therapy and despite the patient’s clinical and biological recovery [10]. The clinical evolution of Pott’s spine is also not parallel to the radiological course [19]. Similarly, a bone marrow high-signal on fat-suppressed T2-weighted images persists 24 months after well-conducted antibiotic therapy for osteitis of the hip bone [20].

It seems interesting to consider our results and previous data in order to optimize the follow-up of patients with spondylodiscitis. Thus, apart from a neurological aggravation, follow-up radiological imaging should not be systematic. Clinical evaluation associated with infectious markers would allow sufficient follow-up to assess the evolution of the disease. However, the accuracy of infectious biomarkers during spondylodiscitis has not been extensively studied. Carragee et al. reported that a 25% decrease in erythrocyte sedimentation rate (ESR) during the first month of antibiotic therapy was rarely associated with management failure [21]. However, the majority of patients without this percentage decrease did not experience therapeutic failure. One previous study showed that C-reactive protein (CRP) was the marker most correlated with soft tissue changes, while ESR was more associated with bone changes [22]. These observations may help in the interpretation of imaging results; thus, in the same way that ESR normalizes more slowly than CRP, it seems that bone abnormalities remain for a longer period than soft tissue abnormalities do. Kowalski et al. suggested that variation of inflammatory biomarkers and the clinical evolution of the patient would allow the selection of patients who actually require follow-up MRI [9,22]. However, there is no ideal marker for follow-up. Thus, an approach based on clinical and biomarkers would make it possible to avoid unnecessary additional expenses and to limit long and sometimes uncomfortable complementary examinations to patients, often elderly [8]. It is interesting to note that in the case of post-surgical PVO, diagnosed by [^18^F]FDG-PET/CT if follow-up imaging is required, the same imaging modality will be preferred. In these situations, the [^18^F]FDG-PET/CT allows the determination of residual infection after treatment [5].

The limitations of our study are mainly based on its retrospective and monocentric nature. The analysis of CT scans is another limitation to be considered. Indeed, CT scans are less sensitive than MRI for analyzing the abnormalities described in our study, especially epiduritis. However, this bias was minimized by comparing the same radiological imaging modality to baseline and during follow-up. Moreover, our study was carried out in a university hospital center that handles cases that are sometimes more complicated or atypical than those encountered in other centers.

## 5. Conclusions

In conclusion, our results support the fact that follow-up radiological imaging during PVO does not correlate with the clinical status of the patient. Its use, apart from proven clinical complications, could complicate the management by providing contradictory data with respect to the clinical observation performed by the physician. Thus, apart from a modification of the clinical examination, notably neurological and aggravation of clinical or biological infectious parameters, follow-up radiological imaging does not seem to be relevant and useful in the follow-up of patients with PVO. Clinical and biological evaluation seems sufficient to determine whether or not the patient is cured. Many images are made during the follow-up with a questionable cost/effectiveness ratio.

## Figures and Tables

**Table 1:** Characteristics of patients.

	Patients *n* = 86
Age, years, mean ± SD	68 ± 13
Sex Ratio M/F	1.5
BMI (kg/cm²)	27.4
Medical History	
Diabetes, *n* (%)	23 (27)
Renal failure *, *n* (%)	12 (14)
PVO, *n* (%)	4 (5)
Spinal surgery, *n* (%)	21 (24)
with material, *n* (%)	9/20 (45)
without material, *n* (%)	12/20 (55)
Drug addiction, *n* (%)	2 (2)
Immunodepression **, *n* (%)	7 (8)
Spinal injury level	
Lumbar	59 (68)
Thoracic	17 (20)
Cervical	10 (12)
Microbiology	
Methicillin-sensitive *Staphylococcus aureus*, *n* (%)	29 (34)
Methicillin-resistant *Staphylococcus aureus*, *n* (%)	4 (5)
Coagulase negative *Staphylococcus, n* (%)	8 (9)
*Staphylococcus epidermidis*, *n* (%)	6 (7)
*Staphylococcus capitis*, *n* (%)	2 (2)
*Streptococcus*, *n* (%)	16 (19)
Gram-negative bacilli, *n* (%)	17 (20)
*Mycobacterium tuberculosis, n* (%)	3 (3)
Others, *n* (%)	9 (10)

* Glomerular filtration rate < 60 mL/min, ** Corticosteroid therapy, biotherapy, chemotherapy, HIV infection, active cancer. BMI: Body Mass Index; PVO: Pyogenic Vertebral Osteomyelitis.

**Table 2:** Clinical, biological, and radiological imaging examinations characteristics.

	At Diagnosis *n* = 86	To the Follow-Up *n* = 86	*p*-Value
Spinal pains, *n* (%)	84 (98)	21 (24)	<0.001
Fever, *n* (%)	64 (74)	4 (5)	<0.001
Neurological impairment, *n* (%)	29 (34)	10 (12)	<0.001
motor deficiency, *n* (%)	5 (5.8)	5 (5.8)	1
medullary compression, *n* (%)	4 (4.7)	1 (1.2)	0.37
radiculalgia, *n* (%)	22 (26)	4 (4.7)	<0.001
C reactive protein, mg/l, mean ± SD	64.9 ± 16	25.9 ± 7	0.01
CT scan, *n* (%)	30 (31) *	30 (31)	
abscesses, *n* (%)	8 (27)	5 (17)	0.35
soft tissue infiltration, *n* (%)	17 (56.7)	9 (30)	0.037
epiduritis, *n* (%)	5 (17)	3 (10)	0.71
MRI, *n* (%)	69 (72) *	69 (72)	
Abscesses, *n* (%)	29 (42)	23 (33)	0.26
Soft tissue infiltration, *n* (%)	56 (81)	38 (55)	<0.01
Epiduritis, *n* (%)	38 (55)	23 (33)	0.01

SD: standard deviation; CT scan: computerized tomography; MRI: Magnetic Resonance Imaging; * Some patients had CT scans and MRIs.

**Table 3:** Follow-up radiological imaging compared to clinical status.

	Clinical Status		
	Cured (*n* = 36)	Unhealed (*n* = 50)	*p*-Value
Mean delay to diagnosis ± SD, days	40 ± 31	48 ± 38	0.72
Follow-up radiological imaging finding, mean ± SD month	2.79 ± 2.2	2.77 ± 2	0.97
Improvement/Stability, *n* (%)	24 (67)	39 (78)	0.68
MRI	12 (50)	24 (62)	0.17
CT scan	12 (50)	15 (38)	0.74
Reduction in soft tissue infiltration, *n* (%)	11 (45)	11 (28)	0.21
Reduction in abscesses, *n* (%)	8 (33)	10 (26)	0.80
Reduction in epiduritis, *n* (%)	8 (33)	11 (28)	0.79
Deterioration, *n* (%)	12 (34)	11 (22)	0.68
MRI, *n* (%)	7 (58)	8 (73)	0.68
Scanner, *n* (%)	5 (42)	3 (27)	0.27
New abscesses, *n* (%)	3 (25)	4 (36)	0.67
Extension of soft tissue infiltration, *n* (%)	2 (17)	1 (9.1)	1
Extension of epiduritis, *n* (%)	2 (17)	3 (27)	0.64
New locations, *n* (%)	1 (8.3)	1 (9.3)	1

CT scan: computerized tomography; MRI: Magnetic Resonance Imaging; SD: Standard Deviation.

## Data Availability

The datasets used and/or analyzed during the current study are available from the corresponding author on reasonable request.
